# An early prediction model for canine chronic kidney disease based on routine clinical laboratory tests

**DOI:** 10.1038/s41598-022-18793-6

**Published:** 2022-08-25

**Authors:** Yiannis Kokkinos, JoAnn Morrison, Richard Bradley, Theodoros Panagiotakos, Jennifer Ogeer, Dennis Chew, Ciaran O’Flynn, Geert De Meyer, Phillip Watson, Ilias Tagkopoulos

**Affiliations:** 1 Process Integration and Predictive Analytics, PIPA LLC, Davis, CA USA; 2 Banfield Pet Hospital, Vancouver, WA USA; 3 WALTHAM Petcare Science Institute, Freeby Lane, Waltham on the Wolds, Melton Mowbray, Leicestershire, LE14 4RT UK; 4 Antech Diagnostics, 17620 Mount Herrmann Street, Fountain Valley, CA USA; 5 College of Veterinary Medicine, Ohio State University, Columbus, OH USA; 6 Department of Computer Science and Genome Center, University of California, Davis, CA USA

**Keywords:** Machine learning, Predictive markers

## Abstract

The aim of this study was to derive a model to predict the risk of dogs developing chronic kidney disease (CKD) using data from electronic health records (EHR) collected during routine veterinary practice. Data from 57,402 dogs were included in the study. Two thirds of the EHRs were used to build the model, which included feature selection and identification of the optimal neural network type and architecture. The remaining unseen EHRs were used to evaluate model performance. The final model was a recurrent neural network with 6 features (creatinine, blood urea nitrogen, urine specific gravity, urine protein, weight, age). Identifying CKD at the time of diagnosis, the model displayed a sensitivity of 91.4% and a specificity of 97.2%. When predicting future risk of CKD, model sensitivity was 68.8% at 1 year, and 44.8% 2 years before diagnosis. Positive predictive value (PPV) varied between 15 and 23% and was influenced by the age of the patient, while the negative predictive value remained above 99% under all tested conditions. While the modest PPV limits its use as a stand-alone diagnostic screening tool, high specificity and NPV make the model particularly effective at identifying patients that will not go on to develop CKD.

## Introduction

Chronic kidney disease (CKD) is a prevalent clinical condition affecting dogs. The incidence of canine CKD has been estimated to be around 0.5–1.0% in the United States^1^, but has been shown to approach 25% in some populations studied, including among breeds with a known predisposition^2,3^. The underlying etiology of CKD in dogs can be wide ranging and difficult to determine during clinical investigation. Dogs with CKD are generally considered to have a worse prognosis and shorter survival times, compared to cats diagnosed with CKD^1,4^. Possible reasons include a shorter life expectancy, especially in larger breed dogs, a faster disease progression in those with protein losing nephropathy because of greater proteinuria, or challenges in achieving optimal medical and nutritional care. This latter point is of particular importance since maintenance of higher body condition scores is associated with better prognoses and longer survival times in dogs with CKD^5^.

Early detection of disease enables a greater opportunity for intervention, generally resulting in better maintenance of quality of life and improved survival times post diagnosis^3,6^. In current practice, diagnosis of canine CKD involves the combined review of biochemical alterations (e.g. elevated creatinine, urea), urinalysis (reduced specific gravity), clinical signs (e.g. weight loss, loss of appetite, increased water consumption and urination), and in some instances supported with diagnostic imaging evidence of abnormalities in renal size and structure. Due to the lack of sensitive diagnostic assays, a single, accurate biomarker to assess renal function in clinical practice does not currently exist^7^, as such azotemia and other features of the disease may be significantly advanced before a diagnosis of CKD is reached.

In recent years there has been growing interest in the diagnostic value that can be leveraged through deep analysis of large sets of health screening data collected as part of routine clinical practice. In human health care, machine learning models have been used to assess risk and inform practice management^8^ and predict individual outcomes^9,10^, length of stay^11^, recommend treatments^12^ and personalized medicine^13,14^. Big data and deep learning strategies therefore offer an opportunity to develop early diagnosis algorithms for CKD. To this end, we have recently described the development and validation of a recurrent neural network (RNN) that leveraged four features from electronic health records (EHR; creatinine, blood urea nitrogen, urine specific gravity, and age) to predict cats at risk of developing CKD before clinical signs of the disease are apparent^15^. Due to the recognition of shortened survival times and worse prognoses in dogs diagnosed with CKD, compared to cats, a study was initiated to determine if a similar modelling approach might also result in a robust and accurate predictive model for canine CKD.

## Methods

### Patient population and CKD prevalence estimation

Data were extracted from electronic health records (EHR) of dogs visiting Banfield Pet Hospitals (Vancouver, WA, USA) between February 1, 1996 and March 31, 2018. At the close of this period, Banfield operated over 1000 hospitals in 42 US states.

### EHR structure, processing, and selection

All data were filtered to include only dogs aged between 1.5 to 22y. Individual EHR included patient information (e.g. date of birth, breed, and sex), and time-stamped patient characteristics (e.g. body weight and reproductive status), blood and urine test results, and clinical information from veterinary examination (diagnosis and unstructured medical notes). In the EHR, diagnosis is a field only filled in when a diagnosis is formally made; further information related to the confirmation of diagnosis quality is outlined in the discussion. Rule-out or disease risk information are handled in other EHR fields. Veterinarians manage the diagnosis field carefully and have the option to update a diagnosis from a previous visit based on subsequent information.

**Figure 1:**
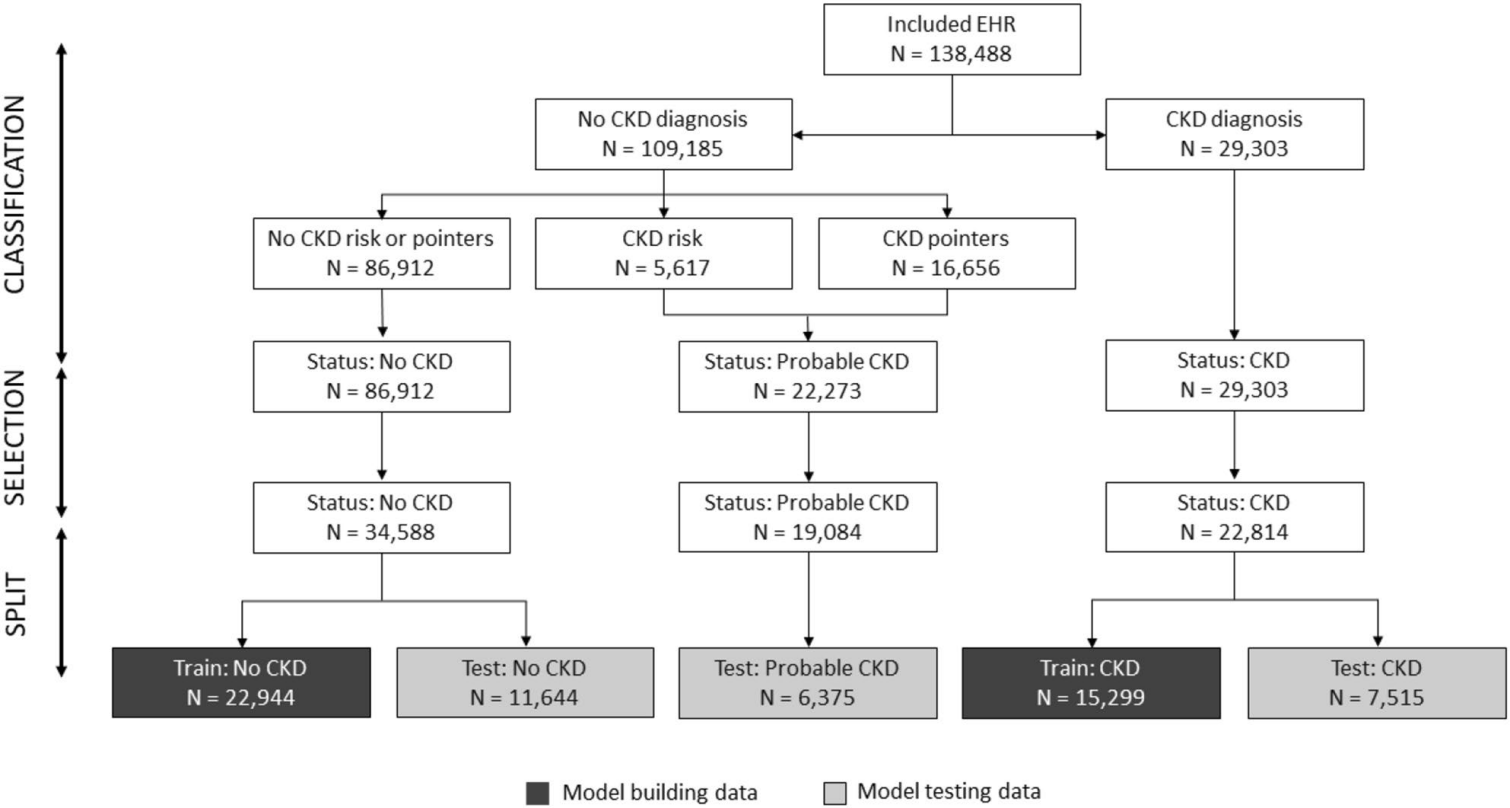
Schematic representation of EHR processing. Starting from the EHR subset with sufficient information for classification (‘Included EHR’) EHR are classified into 3 CKD status groups based on clinical diagnosis, risk and other pointers for CKD in the record. Subsequently records with sufficient visits and blood/urine data prior to the evaluation visit are selected. Finally the EHR are split randomly in training and test sets. For the ‘CKD probable’ status group the training set is not shown as it was not used in the study. EHR, electronic health record.

**Figure 2:**
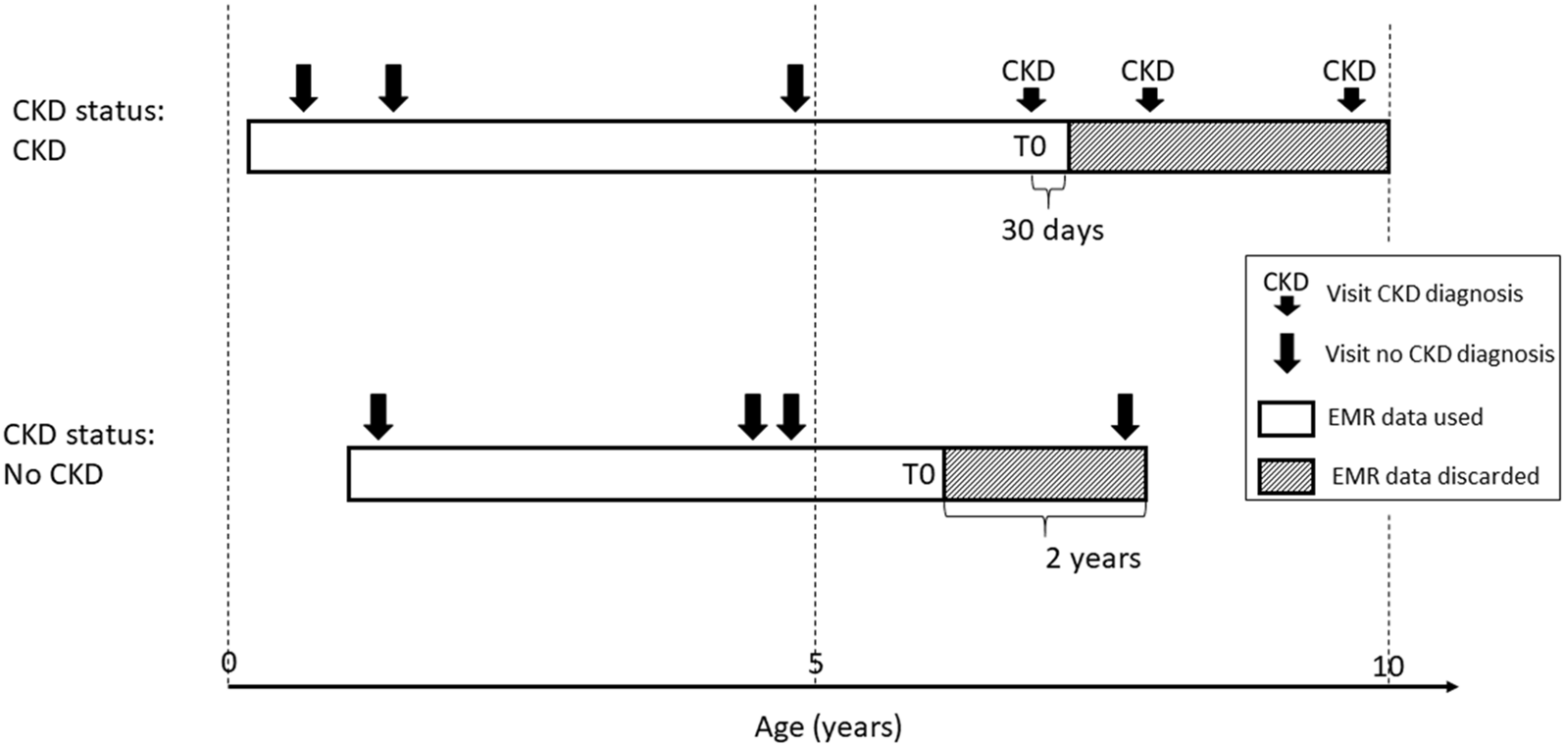
Schematic representation of CKD status assignment, EHR data use, and reference time T0 scaling for 3 hypothetical dog EHR profiles. CKD, chronic kidney disease; EHR, electronic health record.

A sequence of EHR processing and selection steps was performed prior to the analysis (Fig. 1). In a first step EHRs were classified into 3 CKD status groups (Fig. 2). Group 1 consists of EHRs with a CKD diagnosis (CKD status “CKD”). The age at the diagnosis visit was used as the age at evaluation (T0). For this group, data collected more than 30 days after the diagnosis was excluded for model building (an additional 30-day window was included to capture serum, blood or urine test data that was entered into the database shortly after the diagnosis visit). EHRs without a CKD diagnosis, but with either an EHR mention of CKD risk or with at least two CKD-suggesting data points from the following list: blood creatinine above normal values, urine-specific gravity (USG) below normal values, and “CKD”, “azotemic”, “Royal Canin Veterinary diet Renal” or “Hill’s prescription diet k/d” in the medical notes were classified as “probable CKD”. While the exact reason for a lack of a CKD diagnosis is uncertain for these EHRs, it is likely that the veterinarian was either unsure about the diagnosis, was awaiting additional diagnostic results, or did not fill in the diagnosis field for procedural reasons. All EHRs that were not included in the two previous groups, and that had at least 2 years of data (recorded visits) at the end of the EHR to validate absence of CKD were assigned a “no CKD” status. For these EHRs age at evaluation (T0) was set as the age at the last visit minus 2 years, and the last 2 years of data were removed from the EHR. EHRs that lacked the information required for this classification were discarded.

In a second step, classified EHRs were filtered based on having sufficient blood and urine data prior to the T0 visit. After setting extreme outliers for blood and urine tests (more than 6 standard deviations above the maximum of the normal range) to missing, and excluding visits preceding the T0 visit that did not have blood and urine analysis, we retained only these EHRs that had at least 2 visits in the 4 years preceding the T0 visit. In addition, retained EHRs were required to have more than 50% blood and more than 25% urine results in the visits within 4 years prior to the T0 visit.

### Model building

The “CKD” and “no CKD” groups were split further with 67% of the data used to build the CKD prediction model and the remaining 33% were used as a test set to evaluate model performance (Fig. 1). From the available EHR 35 data types, including hematology, clinical chemistry, urinalysis and patient characteristics were selected as features for the CKD prediction model. Prior to the analysis, missing information in the blood and urine test data were imputed using MissForest (v1.416)^16^ in R (v3.4.3)^17^. Imputation used all available blood and urine data but not the CKD status.

The model building approach employed was informed by work previously undertaken to develop an algorithm to predict future CKD diagnosis in cats^15^. In a standard recurrent neural network (RNN) the input feature data at every visit are combined in nonlinear ways through layers and nodes, respectively, and merged with data from previous visits to yield a probability score. To direct the model to perform optimally for early detection of CKD, the model building data set was augmented by adding truncated versions of the original EHRs (last *k* visits removed with *k* ranging from 1 to the total number of visits—1). This enriched the data set with EHRs having a gap of up to 2 years between the last visit seen by the model and the time of diagnosis.

Feature selection was conducted by a top-down and bottom-up wrapper method^18^ using a standard recurrent neural network (RNN^19^) using Tensorflow (v1.3)^20^ and Keras Deep Learning library (v2.0.8)^21^ with a 3–7 hidden layer structure based on the results obtained for cat CKD^15^. The RNN was implemented with a tanh activation function in the hidden layers and softmax for transforming the output layer into a CKD probability score. Backpropagation through time was used for training with the RMSprop gradient optimization algorithm. Model performance was evaluated based on the F1 cross-entropy in a threefold cross-validation setup. We used the F1 cross-entropy as a metric because it balances sensitivity and specificity independent of CKD incidence.

Next a full model architecture screen was performed with the selected features. Different RNN configurations of 1 to 5 hidden layers were tested with 3 to 200 nodes per layer. The setup was the same as above except that 20% dropout was added to avoid overfitting^22^. Evaluation was based on the F1 score in a tenfold cross-validation setup^23^. Finally, the best model configuration was fine-tuned with respect to the training time in the same cross-validation set-up.

### Model testing

Model performance was assessed by applying the selected prediction model to the hold-out test data. Results were interpreted at the level of the crude model output—the probability of a CKD (*p)*—as well as after categorisation into “no CKD” and “CKD” using *p* = 0.5 as the cut-off point. Categorical results for “CKD” and “no CKD” groups were used to compute sensitivity (proportion of true positives, “CKD” status predicted as CKD), specificity (proportion of true negatives, “no CKD” predicted as no CKD) and accuracy (proportion predicted correctly out of the total) estimates. These data enabled the determination of the positive predictive value (PPV = (sensitivity × prevalence)/[(sensitivity × prevalence) + ((1 – specificity) × (1 – prevalence))]) and negative predictive value NPV = (specificity × (1 – prevalence))/[(specificity x (1 – prevalence)) + ((1 – sensitivity) × prevalence)]^24^. Confidence intervals for sensitivity and specificity estimates were calculated using the normal approximation and represent the uncertainty due to the number of data points available. The ability for the model to predict CKD ahead of the definitive diagnosis was evaluated by truncating the test data EHRs to various time points before age at evaluation (T0) for the “CKD” group and allowing the model to only see the truncated data.

## Results

### Study data set and clinical CKD diagnosis

The study contained dogs that visited a Banfield Pet Hospital between 1996 and 2018 and included both mixed and pedigree breeds. The total data set included records for 6.5 million dogs between 1.5 and 22 years old with at least 2 visits. Within this initial data set 54,098 dogs received a CKD diagnosis which gave an approximate disease prevalence of 0.83%. After the second filtering step, for visits preceding T0 and completeness of blood and urine data, a study population of 57,402 individual dogs with high quality EHRs was created (Fig. 1).

Demographics of this sample differentiated by CKD status, and summaries of blood and urine test data at the time of evaluation (T0), are shown in Table 1. The occurrence of missing data was approximately 10% for most of the blood chemistry measures and up to 60% for urine test results, which are not typically routinely measured at every visit.

**Table 1:** Demographics and summaries for the study data set at the time of evaluation (T0).

Dataset	Training	Training	Test	Test
Diagnosis group	CKD	No CKD	CKD	No CKD
Number of dogs	15,299	22,944	7515	11,644
Mean visits per dog	15.18	11.99	15.22	11.91
Male to female ratio	1:1.1	1:0.93	1:1.1	1:0.94
Mean (SD) age (years) at T0	11.56 (3.37)	7.19 (2.93)	11.59 (3.37)	7.12 (2.90)
Mean (SD) weight (kg) at T0	13.04 (11.20)	15.10 (12.42)	13.34 (11.32)	14.81 (12.11)
Mean (SD) BUN (mg/dL) at T0	56.23 (32.72)	17.38 (5.59)	55.86 (32.80)	17.50 (5.78)
Mean (SD) creatinine (mg/dL) at T0	2.67 (1.86)	1.09 (0.29)	2.67 (1.88)	1.09 (0.27)
Mean (SD) urine protein (mg/dL) at T0	91.01 (180.81)	49.52 (136.21)	94.29 (190.89)	48.61 (146.51)
Mean (SD) USG at T0	1.020 (0.011)	1.039 (0.012)	1.020 (0.011)	1.039 (0.012)
Percent missing creatinine	11.32%	7.26%	10.91%	7.72%
Percent missing USG	57.52%	60.83%	57.58%	60.89%

Mean and Standard Deviation (SD) are shown for continuous measures. USG, urine-specific gravity; BUN, blood urea nitrogen.

**Figure 3:**
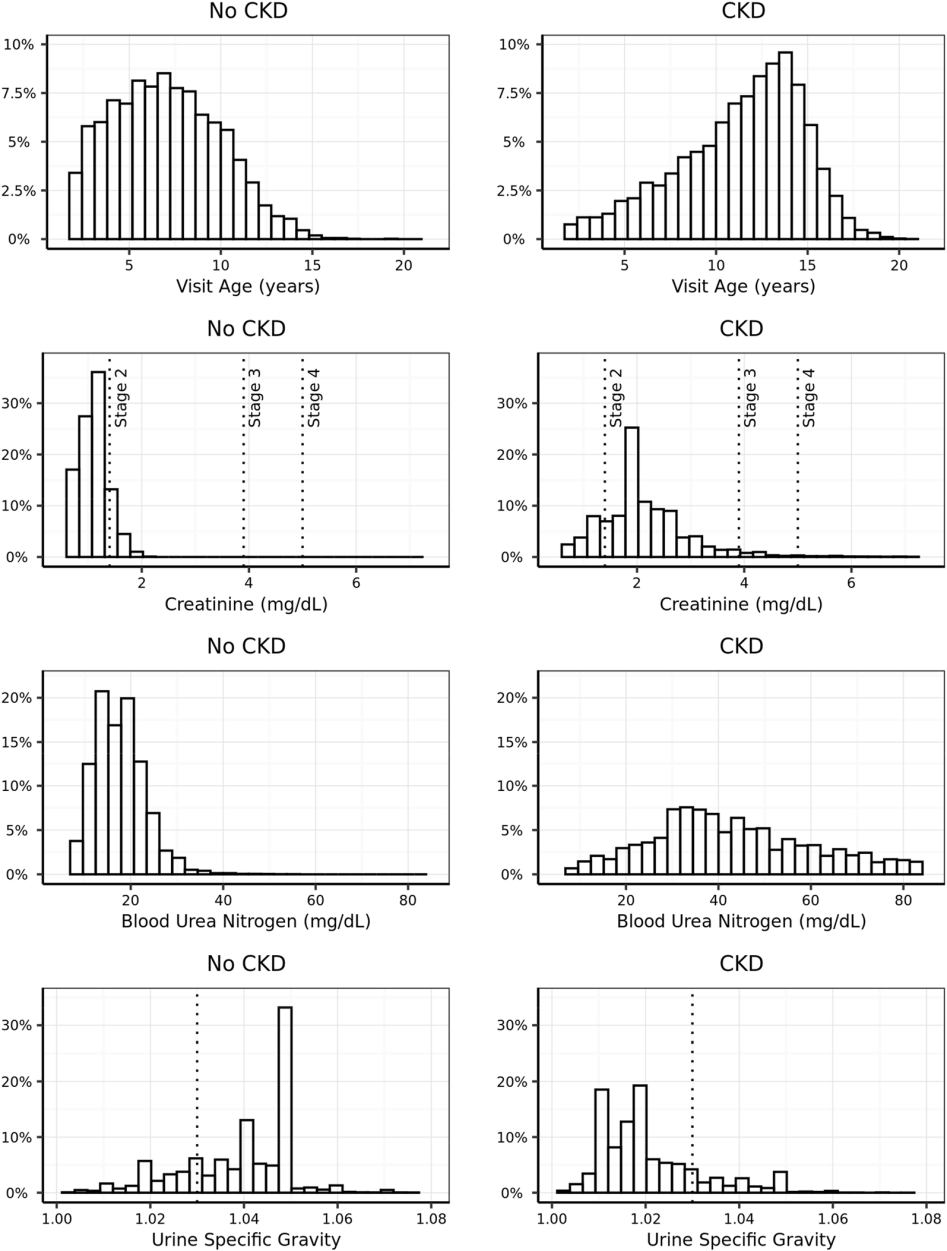
Distribution of age, creatinine, BUN and USG at the time of evaluation (T0) in the study EHR set differentiated by CKD status. Dotted lines on the creatinine line and USG plots show limits used for staging in the IRIS guidelines. CKD, chronic kidney disease; IRIS, International Renal Interest Society; USG, urine-specific gravity; BUN, blood urea nitrogen.

As multiple guidelines for the diagnosis of CKD exist, and these might have evolved during the period captured in this study, we explored how the CKD status as used in this study relates to various diagnostic measurements routinely used when making CKD diagnoses. Dogs with status “CKD” were generally older, had higher creatinine levels, reflected by distribution of “CKD” and “no CKD” dogs versus the International Renal Interest Society (IRIS) staging boundaries, and lower USG compared to dogs with “no CKD” status (Fig. 3). For all measurements there was an overlap in the distributions between CKD status groups, such that any single parameter alone does not have sufficient discriminatory power for diagnosis. This intrinsically multifactorial nature of canine CKD presents an ideal setting for prediction models to add clinical value.

### Building a prediction model for CKD

We used a standard RNN with a 3–7 hidden layer structure as a starting point for a prediction model for CKD that acknowledges both the multifactorial and temporal aspects of CKD development. Using this type of model with 35 features was impractical both for training the model as well as for using it in practice later. Therefore, we first set out to select the most important features using a top-down and bottom-up feature selection strategy on the training data set. This approach showed that model performance in terms of the cross-entropy score improved by adding up to 6 features and plateaued thereafter (data not shown). The best model feature set was determined to be creatinine, blood urea nitrogen (BUN), urine specific gravity (USG), urine protein, weight, and age. With these 6 features we determined the best model to be a three-layer RNN with a 5-3-3 structure trained over 8 epochs.

### Detecting CKD at the time of diagnosis

The performance of the CKD model at the time of diagnosis was calculated using the 15,152 EHRs which had a visit within 3 months of diagnosis, out of the 19,159 EHRs in the test data set. Categorisation of this result with a threshold at *p* = 0.5 resulted in a sensitivity of 91.4% (9955/10,889) based on the status “CKD” and a specificity of 97.2% (4142/4263) based on the status “no CKD”.

**Figure 4:**
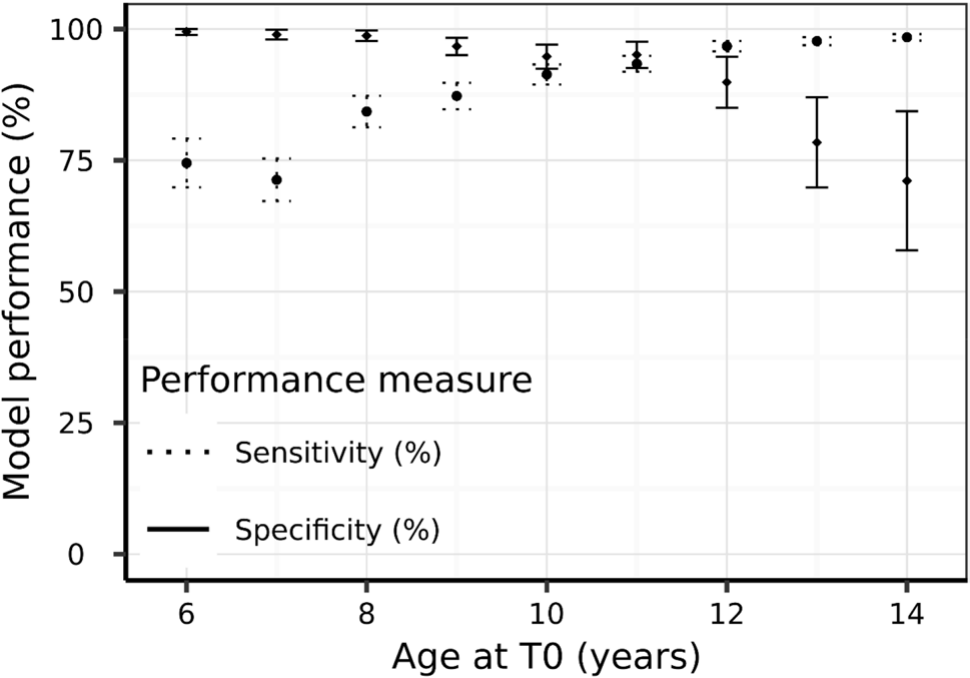
Model sensitivity and specificity with 95% confidence intervals as a function of age at evaluation.

As age is a model feature sensitivity and specificity were also reported by age at evaluation (T0) in Fig. 4. Specificity was consistently above 98% until an age of 6 years and declined thereafter reaching 67% for an age of 15 years. Sensitivity increases with the age at T0 and is over 96% from 12 years old onwards. Therefore, the model sacrifices some specificity (increased false positive rate) for better sensitivity (lower false negative rate) when predicting older pets, where CKD prevalence is higher. This dynamic results in a positive predictive value ranging from 15 to 23% across age groups (Table 2).

**Table 2:** CKD prevalence, model sensitivity, specificity, positive predictive value and negative predictive value estimates for CKD prediction at the time of diagnosis for different life stage groups.

Life stage	Sensitivity (%)	Specificity (%)	Prevalence (%)	PPV (%)	NPV(%)
Adult	62.42	99.69	0.15	22.86	99.94
Mature	82.40	97.99	0.48	16.57	99.91
Senior	94.99	92.88	1.46	16.47	99.92
Geriatric	98.90	69.52	5.21	15.14	99.91

Adult is defined as 1.5 to 6.5 years, mature as 6.5 to 9.75 years and under, senior 9.75 to 13 years, and geriatric as over 13 years. Disease prevalence is estimated from the life stage groups within the study population and model performance determined using the test data set.

**Figure 5:**
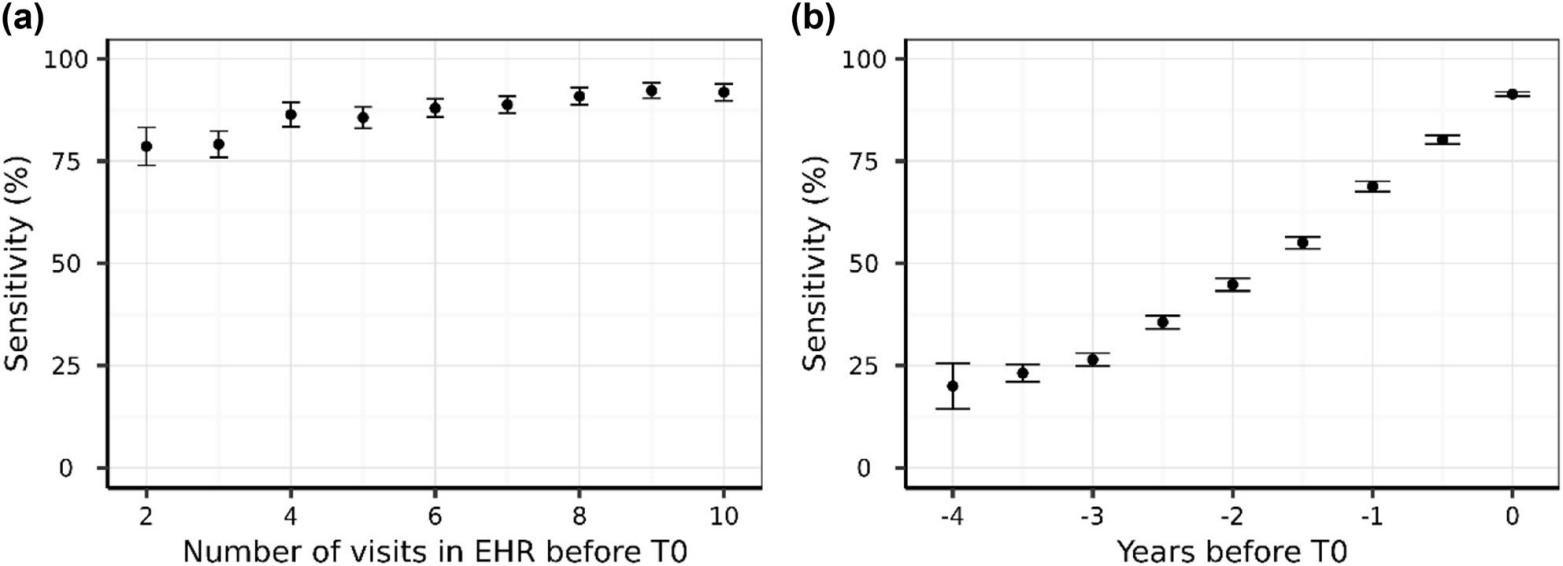
Model sensitivity at T0 with 95% confidence intervals as a function of the number of visits in the EHR before the time of diagnosis T0 (**a**) and model sensitivity with 95% confidence intervals as a function of the time before diagnosis where the prediction was made only with the data up to that point (**b**).

To understand how the patient history affected model performance we examined model sensitivity as a function of the number of visits in the EHR before the diagnosis was made. Sensitivity increases from 78.6% with 2 visits, to 86.4% with 4 visits prior to the diagnosis, and continues to improve to over 92% with further data (Fig. 5a). This shows that longitudinal information significantly contributes to the quality of the CKD diagnosis.

### Using the model for early detection

We evaluated the model’s ability to predict a future CKD diagnosis (Fig. 5b and Table 3). As expected, sensitivity decreased when increasing the time between prediction and diagnosis, although of the dogs that went on to develop CKD, 68.8% were correctly predicted 1 year before diagnosis, 44.8% 2 years before diagnosis, and 23.2% as far as 3.5 years prior to clinical diagnosis.

**Table 3:** Model predictive performance metrics.

Years before T0	Sensitivity	Specificity	Accuracy	PPV	NPV
0	91.42	97.16	93.04	21.22	99.93
0.5	80.22	97.63	90.34	22.07	99.83
1	68.79	98.18	84.83	24.02	99.73
1.5	55.00	98.51	78.79	23.59	99.62
2	44.76	98.95	73.03	26.29	99.54
2.5	35.64	99.36	68.62	31.78	99.46
3	26.48	99.49	61.81	30.28	99.39
3.5	23.20	99.65	59.85	35.67	99.36

For all PPV and NPV calculation the prevalence was fixed at 0.83% (overall data set prevalence).

For dogs in the “no CKD” category there is, by definition, an absence of a CKD diagnosis, but for the purpose of model building and evaluation T0 for a “no CKD” dog was assigned as 2 years prior to their most recent database record (Fig. 2), contrary to the definition for “CKD” dogs which was the point of clinical diagnoses. Assessing specificity, the ability to detect dogs without CKD, within the context of a trajectory towards T0 does not make sense as T0 bears no clinical temporal relevance. The specificity for early CKD detection is instead best appreciated by its distribution with age where the average specificity remains above 90% for dogs between 4 and 12 years old.

The PPV of the test ranged between 21 and 36% when predicting future risk of CKD, whereas the NPV remained above 99% at all calculated time horizons to diagnosis (Table 3).

## Discussion

In human healthcare longitudinal patient data and deep learning approaches have recently been applied to build models to diagnose CKD^25^, predict future risk of acute kidney injury^26^ and to differentiate patients with CKD that are more likely to progress^27^. We have recently described the development and validation of a recurrent neural network (RNN) that leveraged four features from EMRs (creatinine, blood urea nitrogen, urine specific gravity, and age) to predict cats at risk of developing CKD before clinical signs of the disease are apparent^15^. Here we applied a similar computational modelling approach to a large set of EHRs from a network of primary care hospitals to derive a model to predict CKD risk in canines at a given point in time based on current and past EHR data. This data, consisting primarily of clinicopathologic results, was evaluated and refined, resulting in a RNN model consisting of six features: serum creatinine, BUN, urine protein and USG, and age and weight of the patient. We then evaluated the performance of this model for predicting the risk of dogs developing CKD in the future.

### Model features and model performance

Of the dogs that went on to develop CKD, 68.8% were correctly predicted 1 year before diagnosis, 44.8% 2 years before diagnosis, and 23.2% as far as 3.5 years prior to clinical diagnosis. Model performance improved with more frequent data collection points, reflected in a sensitivity of 78.6% with data from two visits, which increased to 86.4% when longitudinal data from four visits was available. There are two likely explanations for this observation. Firstly, simply having more data available enables the algorithm to make a better prediction. It is also possible that suspicion of an underlying condition by the veterinarian leads to more regular testing, which in turn leads to more data and consequently an improved prediction. Specificity increased to over 95% with additional data. The model also performed well in different age categories of dogs, where specificity was consistently maintained above 90% in dogs within adult (1.5–6.5 y), mature (6.5–9.75 y) and senior (9.75–13.0 y) life stages. As dogs aged further, the specificity declined to 70% for dogs in geriatric life stage (over 13 years). Conversely, the sensitivity of the prediction was improved in older dogs, with a sensitivity of 99% in dogs of 13 years and older. In older pets, it is more common to have multiple diagnoses and co-morbid conditions. Here we observed that dogs with nuclear sclerosis and osteoarthritis were more likely to have a false positive result, perhaps due to the strong age association in the prevalence of these conditions. It is therefore a benefit that this model sacrifices some specificity (which slightly increases the false positive rate) for a better sensitivity (which lowers the false negative rate) in older pets. In practice, this will help ensure that the clinician does not overlook the presence of CKD in an older pet.

### Clinical utility

The prevalence of disease in the population is particularly important when considering the clinical utility of any diagnostic tool. While sensitivity and specificity are not influenced by disease prevalence, the positive (PPV) and negative (NPV) predictive values are, and become particularly relevant in accounting for differences within the test population. Compared to cats, dogs typically display a low prevalence of CKD (Table 2) and it is important to recognise the impact this will have on the true diagnostic accuracy of the algorithm. Compared to the application of a similar modelling approach in cats, where the PPV remained above 90% under all tested conditions^15^, here the PPV was highest when predicting future risk of dogs aged 6.5y and under (23%), but was reduced to 15% in the oldest dogs included in the test data set. The PPV is useful to the clinician as it indicates the likelihood of disease in a patient when the test result is positive, but it is important to not discount the value of a tool that displays lower PPV values^28^. Interestingly the model displayed consistently high specificity (97–99%) and NPV (> 99%), indicating that a negative test result will accurately predict no diagnosis of CKD in dogs up to 3.5 years into the future, with a very low false positive rate. With this in mind, the present approach could be effectively employed to support proactive wellness initiatives, where the goal is to provide confidence that the pet will remain healthy. Furthermore, it is important to note that population prevalence of canine CKD remains a latent variable in our context. We have estimated prevalence using the EHR data available to us, and this aligns with other estimates previously reported^1^ but may still over or under account for the true prevalence of canine CKD within the whole population or within particular at risk subpopulations of dogs.

When using EHRs to enable the development of an algorithm to predict future disease, confirming the accuracy of the diagnosis was an important first step. Data used to build and validate this model came from a very large number of established clinics and a broad range of veterinarians collated over a period of more than 20 years. Dogs with a formal CKD diagnosis showed blood parameters and urine patterns that are consistent with currently accepted guidelines, with most dogs in the CKD group displaying creatinine values consistent with IRIS stage 2 and 3 (Fig. 3); this provides confidence in the use of these data to develop the model. The definition of the health status of the complementary set of dogs without a formal CKD diagnosis was more problematic. A subset of these, those that were classified as “probable CKD” had clear indications for CKD in blood or urine test results or references in the medical notes that suggest CKD. This group of dogs includes those where the veterinarian was unsure of the diagnosis (most likely because of conflicting information) or because the dog was in an early stage of the disease, or where for unknown reasons they could not be diagnosed. Whereas case‐control studies typically exclude these somewhat ambiguous patients, thus creating a wider space between the groups and enhancing the statistical significance of findings, we decided that the inclusion of these during the testing phase was important to ensure the predictive capability of the algorithm was tested in a real-world scenario where ambiguous cases are present. We did not include this group when computing sensitivity, however, and we are aware that this could bias our estimates given that it could contain the more difficult cases to predict. For the other dogs without a formal CKD diagnosis, we imposed a 2‐year window with observations to be confident of their “no CKD” status. This could have reduced our specificity estimates as some might have had very early-stage CKD that was diagnosed more than 2 years later.

While serum creatinine is widely referenced as a diagnostic marker of CKD, and a surrogate indicator of changes in glomerular filtration rate (GFR), it has limitations and should be applied cautiously especially during times of lean muscle mass loss. It is possible that part of the deficiency in the application of creatinine to CKD may be improved through the integration of body weight and age within the algorithm. In human medicine, clearance methods to measure glomerular filtration rate (GFR) are routinely undertaken in the diagnosis of CKD, and repeated measures of GFR within the same individual are typically used to determine treatment effects and disease prognosis. Additionally, statistical approaches to estimate GFR (eGFR) have been validated and deployed in clinical settings to track progression of patients with CKD. Determining GFR in a primary care veterinary setting is not feasible and to our knowledge, a validated eGFR has not been successfully developed in companion animals. A range of prediction models have been developed to support the diagnosis and progression of kidney disease in humans^25–27,29^. Predicting patients at risk of CKD progression can help guide individualized treatment plans, and this has been shown to be particularly effective at improving outcomes for those at risk of kidney failure requiring dialysis or transplant in human patients^29^. The present model is not able to distinguish between patients where the disease will progress, and those that will not, and further work to improve the capabilities of the predictive algorithm in this way would be beneficial.

In conclusion, CKD in dogs remains a challenging clinical condition with a shorter survival time compared with cats. When diagnosed, the disease is often advanced (IRIS stage 3 or 4), and unlike cats receiving appropriate management, typically progresses more rapidly with poor clinical outcome. The development of tools that can enable veterinarians to identify dogs at risk of CKD prior to the presence of clinical signs opens opportunities to take a significant step forward in the management of this condition. The main goal of the present study was to develop a method that allows a prediction for a future diagnosis of CKD in dogs, but the obvious question to be asked next is how best to support patients with a positive prediction. Here we believe that close monitoring for the development of proteinuria and assessment of blood pressure is critical, as both renal proteinuria and systemic hypertension are risk factors in the progression of CKD in dogs^3,4^. It would also be prudent to recommend other management tactics, including appropriate nutritional interventions^6^, and to avoid or use extreme caution with the use of drugs that are potentially nephrotoxic, but further studies are required to validate the effectiveness of such interventions at improving clinical outcome. While the sensitivity and specificity of the present model was similar to the performance of an algorithm designed to predict future risk of CKD in cats, the modest PPV means that its utility as a standalone screening tool today may be limited. However, this algorithm displayed high specificity and NPV, making it particularly effective at identifying patients that will not go on to develop CKD. Taken together, we believe this approach has the potential to complement existing clinical suspicion and diagnostic investigations of patients, thereby supporting the practitioner’s decision-making process.
